# Glutathione peroxidase 4 suppresses manganese-dependent oxidative stress to reduce colorectal tumorigenesis

**DOI:** 10.21203/rs.3.rs-3837925/v1

**Published:** 2024-01-08

**Authors:** Xiang Xue, Zhaoli Liu, Yanshan Liang, Young-Yon Kwon, Rui Liu, David Martin, Sheng Hui

**Affiliations:** University of New Mexico; University of New Mexico; Harvard T.H. Chan School of Public Health; Harvard T.H. Chan School of Public Health; University of New Mexico; UNM; Harvard

**Keywords:** Colorectal cancer, Oxidative stress, Experimental Colitis, Cell Death

## Abstract

The role of glutathione peroxidase 4 (GPX4) in ferroptosis and various cancers is well-established; however, its specific contribution to colorectal cancer has been unclear. Surprisingly, in a genetic mouse model of colon tumors, the deletion of GPX4 specifically in colon epithelial cells increased tumor burden but decreased oxidized glutathione. Notably, this specific GPX4 deletion did not enhance susceptibility to dextran sodium sulfate (DSS)-induced colitis in mice with varied iron diets but showed vulnerability in mice with a vitamin E-deficient diet. Additionally, a high manganese diet heightened susceptibility, while a low manganese diet reduced DSS-induced colitis in colon epithelial-specific GPX4-deficient mice. Strikingly, the low manganese diet also significantly reduced colorectal cancer formation in both colon epithelial-specific GPX4-deficient and wildtype mice. Mechanistically, antioxidant proteins, especially manganese-dependent superoxide dismutase (MnSOD or SOD2), correlated with disease severity. Treatment with tempol, a superoxide dismutase mimetic radical scavenger, suppressed GPX4 deficiency-induced colorectal tumors. In conclusion, the study elucidates the critical role of GPX4 in inhibiting colorectal cancer progression by regulating oxidative stress in a manganese-dependent manner. The findings underscore the intricate interactions between GPX4, dietary factors, and their collective influence on colorectal cancer development, providing potential insights for personalized therapeutic strategies.

## Introduction

Ferroptosis, a distinctive process among various types of cell death, has garnered significant attention in cancer research. It is a mechanism through which cells, particularly cancer cells, undergo elimination due to the accumulation of excessive iron and lipid peroxides (Stockwell et al. 2017). Ferroptosis can be considered an intrinsic defense mechanism against cancer, especially given the heightened vulnerability of many cancer cells to ferroptosis due to their elevated metabolic rate and increased demand for iron and lipids. Consequently, inducing ferroptosis in cancer cells emerges as a promising therapeutic strategy.

While ferroptosis holds promise as a cancer treatment strategy, several challenges persist. For example, colorectal cancer, which accumulates substantial iron, has developed mechanisms to resist ferroptosis. Notably, the expression of glutathione peroxidase 4 (GPX4), a key protective protein that reduces lipid peroxides and prevents ferroptosis, is upregulated in colorectal cancer patients undergoing neoadjuvant chemoradiotherapy, correlating with a poor prognosis (Zhang et al. 2022).

The resistance of colorectal cancer to ferroptosis poses a challenge in cancer treatment. Researchers are actively exploring ways to exploit ferroptosis as a therapeutic approach. Investigations involve the use of small molecules and drugs that either inhibit GPX4 or induce lipid peroxidation to selectively target cancer cells. Additionally, there is ongoing research into combining ferroptosis-inducing agents with traditional cancer therapies, such as chemotherapy or radiation.

A comprehensive understanding of the precise mechanisms underlying ferroptosis in cancer cells and the development of effective and safe therapies are ongoing areas of research. For example, a study demonstrated that the inhibition of serine/arginine-rich splicing factor 9 (SRSF9) enhances the sensitivity of colorectal cancer to erastin-induced ferroptosis by reducing GPX4 expression (Wang et al., 2021). In a separate study, sodium butyrate was found to promote ferroptosis by inducing lipid reactive oxygen species (ROS) production through the downregulation of GPX4, leading to reduced tumor growth in xenografts and a colitis-associated colorectal tumor model (Wang et al., 2023). Furthermore, the GPX4 inhibitor RSL3 was shown to hinder colorectal cancer cell growth by inducing ferroptosis, an effect reversible by overexpressing GPX4 (Sui et al., 2018). Moreover, the deficiency of GPX4 in myeloid cells has been demonstrated to actively promote the progression of colorectal cancer (Canli et al., 2017). Specifically, this deficiency leads to an augmented production of ROS, instigating genome-wide DNA mutations within intestinal epithelial cells, ultimately fostering tumor invasion. However, no studies have investigated the influence of colon epithelial-specific GPX4 knockout on colorectal cancer progression.

Our findings reveal that GPX4 depletion in colorectal epithelial cells unexpectedly enhances colorectal tumorigenesis. Mechanistically, under conditions of high oxidative stress, including vitamin E deficiency, elevated manganese (Mn) exposure, and tumor development, superoxide dismutase (SOD2) is upregulated in the colon tissues of colorectal epithelial GPX4 deficient mice. A low Mn diet and the SOD mimetic radical scavenger Tempol mitigate GPX4 deficiency induced SOD2 expression and colorectal tumor formation. These findings underscore the crucial role of GPX4 in suppressing Mn-dependent oxidative stress to curtail colorectal tumorigenesis.

## RESULTS

### Depletion of GPX4 specifically in colon epithelial cells promotes colorectal tumorigenesis.

**Figure 1:**
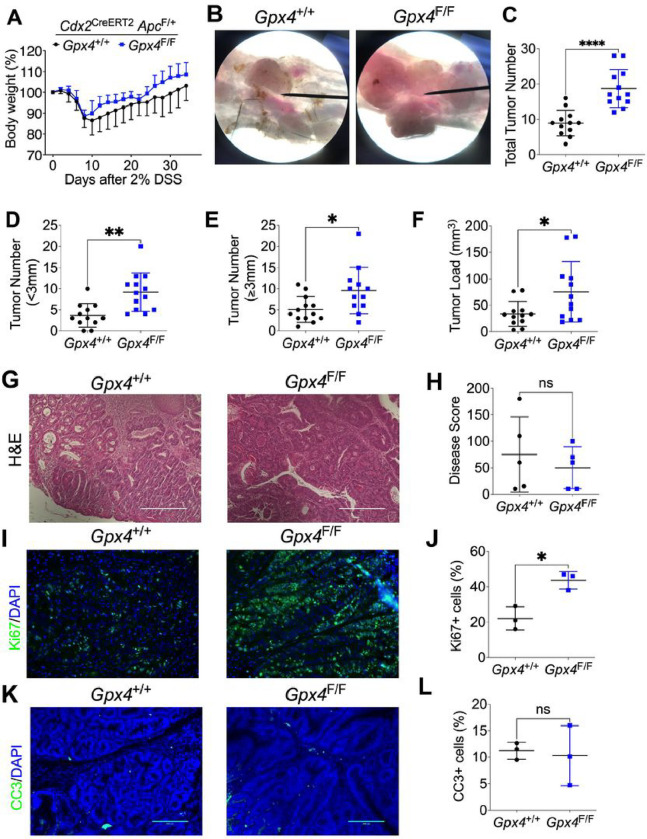
GPX4 depletion specifically in colon epithelial cells promotes colorectal tumorigenesis. (**A**) Body weight curve, (**B**) macroscopic colorectal tumor images, (**C**) total tumor number, (**D**) tumor number for diameter less than 3mm, (**E**) tumor number for diameter more than 3mm. (**F**) Tumor loading, (**G**) H&E staining and (**H**) pathological score, (**I**) Ki67 staining and (**J**) its quantification. (**K**) Cleaved caspase 3 (CC3) staining in colorectal tumor from in *Cdx2* ^CreERT2^ *Apc*^*F/+*^ (n=12) and *Cdx2* ^CreERT2^ *Apc*^*F/+*^ *Gpx4*^F/F^ mice (n=12) and (**L**) its quantification. *p < 0.05, **p < 0.01, ****p < 0.0001, NS not significant. Unpaired Student t test.

To investigate the role of GPX4 in colorectal tumorigenesis, we generated mice with colon epithelial cell specific *Gpx4* depletion. GPX4 deletion was confirmed by genotyping (**Figure S1A**) and qPCR (**Figure S1B**). Colorectal cancer was induced as described in the methods, with the treatment scheme illustrated in **Figure S1C**. We observed that, compared to *Cdx2* ^CreERT2^ *Apc*^*F/+*^ mice, mice with colon epithelial-specific GPX4 depletion (*Cdx2* ^CreERT2^ *Apc*^*F/+*^ *Gpx4*^F/F^) showed no difference in body weight change compared with GPX4 wildtype mice (Fig. 1A). However, GPX4 deficient mice exhibited increased colorectal tumor formation under a dissection microscope (Fig. 1B). The number of total tumors (Fig. 1C), tumors with a diameter less than 3mm (Fig. 1D), and tumors with a diameter more than 3mm (Fig. 1E) were all increased in the GPX4 deficient mice. Additionally, there was an increased tumor burden in the GPX4 deficient mice (Fig. 1F). H&E staining of colorectal tumors from GPX4 deficient and wildtype mice (Fig. 1G) indicated a similar disease score in both groups (Fig. 1H). Ki67 staining (Fig. 1I), and quantification (Fig. 1J) suggested increased cell proliferation in the GPX4 deficient mice, while the apoptosis marker CC3 was not changed (Fig. 1K and 1L). These results suggest a tumor-inhibiting function of GPX4 in colorectal cancer.

### Depletion of GPX4 specifically in colon epithelial cells results in reduced oxidized glutathione levels in colorectal tumors.

**Figure 2:**
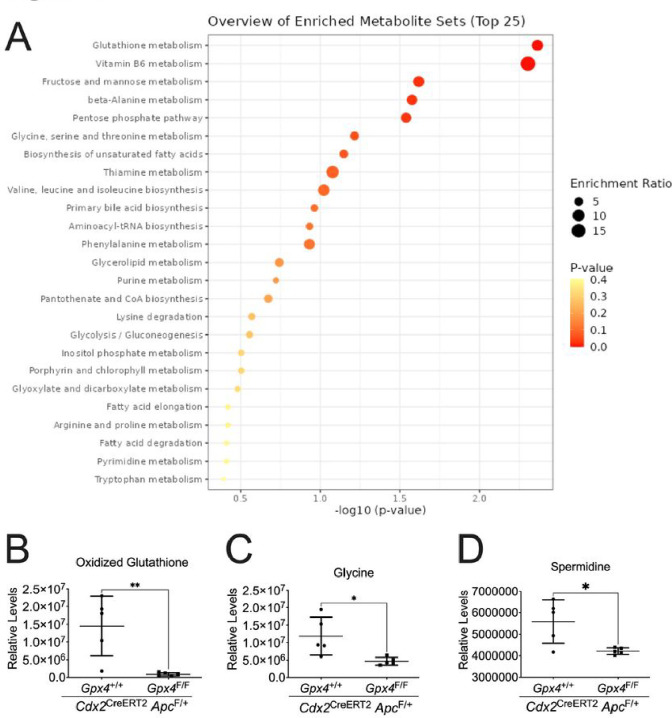
GPX4 depletion specifically in colon epithelial cells leads to redox imbalance. (**A**) Overview of enriched Top 25 metabolites sets in colon tumors from mice with GPX4 depletion specifically in colon epithelial cells. The levels of (**B**) reduced oxidized glutathione, (**C**) glycine and (**D**) spermidine in colon tissues from *Cdx2* ^CreERT2^ *Apc*^*F/+*^ (n=5) and *Cdx2* ^CreERT2^ *Apc*^*F/+*^ *Gpx4*^F/F^ mice (n=5). *p < 0.05, **p < 0.01, NS not significant. Unpaired Student t test.

To understand the mechanism behind the enhanced colorectal tumorigenesis in GPX4-deficient mice, we conducted metabolomics analysis on colon tumors from *Cdx2* ^CreERT2^ *Apc*^*F/+*^ *Gpx4*^F/F^ mice and *Cdx2* ^CreERT2^ *Apc*^*F/+*^ mice. Targeted metabolomics revealed two significantly changed metabolites (**Table S2**): Threonine and Homovanillic acid. In untargeted metabolomics, 41 significantly changed metabolites were identified in the negative mode and 85 in the positive mode (**Table S2**). Enrichment analysis of these 127 significantly changed metabolites using MetaboAnalyst 6.0 highlighted glutathione metabolism and Vitamin B6 metabolism as the top two enriched metabolite sets (Fig. 2A). This enrichment is due to decreased levels of oxidized glutathione (Fig. 2B), glycine (Fig. 2C), and spermidine (Fig. 2D) in the colon tumors of *Cdx2* ^CreERT2^ *Apc*^*F/+*^ *Gpx4*^F/F^ mice compared to those in *Cdx2* ^CreERT2^ *Apc*^*F/+*^ mice. These findings suggest a redox imbalance status due to the critical role of GPX4 in glutathione oxidation.

### GPX4 depletion specifically in colon epithelial cells increases susceptibility to colitis in mice treated with a vitamin E-deficient diet.

**Figure 3:**
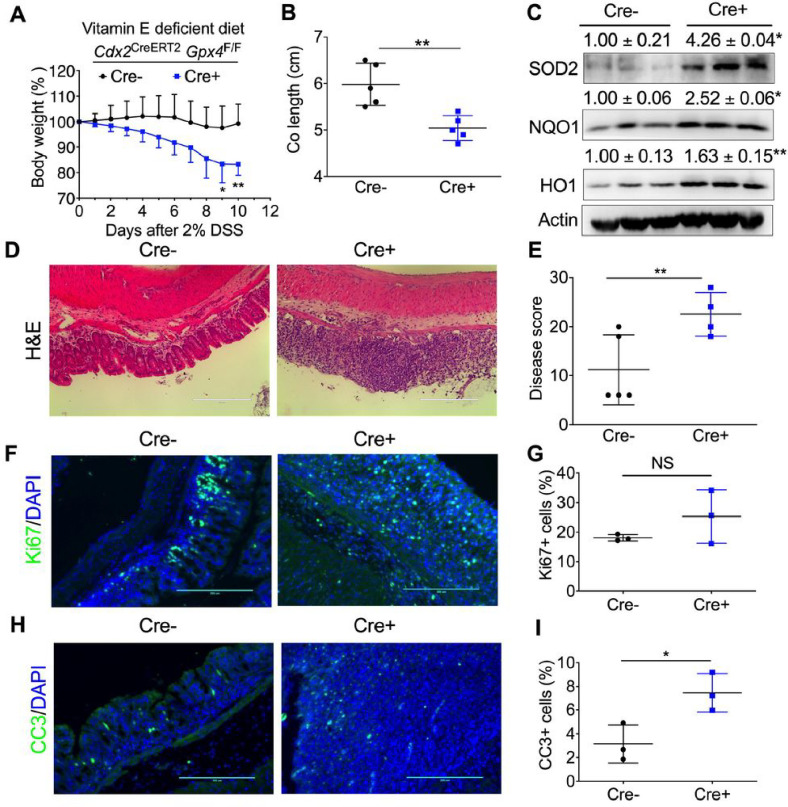
GPX4 depletion specifically in colon epithelial cells leads to increased intestinal inflammation when treated with vitamin E deficient diet. (**A**) Body weight loss curve, (**B**) colon length, (**C**) Western blot analysis of antioxidant protein levels of NQO1, HO-1, and SOD2, (**D**) H&E staining of colon and (**E**) its disease score, (**F**) Ki67 staining and (**G**) its quantification, (**H**) CC3 staining and (**I**) its quantification in colon tissues from *Gpx4*^F/F^ (Cre-, n=5) and *Cdx2* ^CreERT2^ *Gpx4*^F/F^ (Cre+, n=5) mice. *p < 0.05, **p < 0.01, NS not significant. Unpaired Student t test.

As inflammation is a high-risk factor for colorectal cancer and intestinal epithelial GPX4 restricts enteritis (Mayr et al, 2020), we investigated whether colon epithelial GPX4 deficiency in mice affects their susceptibility to colitis. There was no difference in body weight and colon length between *Cdx2* ^CreERT2^ *Gpx4*^F/F^ mice and *Gpx4*^F/F^ mice after treating mice with tamoxifen and DSS. Despite GPX4 being a well-known ferroptosis inhibitor, no differences were observed in body weight and colon length between *Cdx2* ^CreERT2^ *Gpx4*^F/F^ mice and *Gpx4*^F/F^ mice with different iron concentrations in their diets (3.5ppm, 40ppm, 1000ppm) (**Figure S2A–S2H**). Similar results were seen after *Salmonella* treatment (**Figure S2I and S2J**). A vitamin E supplement diet has been demonstrated to reduce GPX4 deficiency-enhanced ferroptosis in hematopoietic stem and progenitor cells (Hu et al. 2021). Interestingly, *Cdx2* ^CreERT2^ *Gpx4*^F/F^ mice exhibited increased body weight loss (Fig. 3A) and shorter colon lengths compared to *Gpx4*^F/F^ mice (Fig. 3B) when induced with DSS and treated with a vitamin E-deficient diet. qPCR analysis indicated that the expression level of *Gpx4* was significantly decreased in the colon tissues of *Cdx2* ^CreERT2^ *Gpx4*^F/F^ mice compared to wildtype control (**Figure S3A**). Interestingly, proinflammatory cytokines including *Tnfa* **(Figure S3B)**, *Il6* **(Figure S3C)**, *Cxcl1* **(Figure S3D)** and *Ptgs2* **(Figure S3E)**, as well as stem cell marker *Lgr5* **(Figure S3F)** was not changed. However, the anti-inflammatory cytokines including Il10 **(Figure S3G)**, *Il22* **(Figure S3H)**, and transcription factor *Foxm1* **(Figure S3I)** were significantly decreased in the colon tissues of *Cdx2* ^CreERT2^ *Gpx4*^F/F^ mice. qPCR analysis revealed a significant decrease in the expression level of *Gpx4* in the colon tissues of *Cdx2* ^CreERT2^ *Gpx4*^F/F^ mice compared to the wildtype control (**Figure S3A**). Interestingly, proinflammatory cytokines, including *Tnfa* (**Figure S3B**), *Il6* (**Figure S3C**), *Cxcl1* (**Figure S3D**), *Ptgs2* (**Figure S3E**), and the stem cell marker *Lgr5* (**Figure S3F**), remained unchanged. However, anti-inflammatory cytokines, including *Il10* (**Figure S3G**), *Il22* (**Figure S3H**), and the transcription factor *Foxm1* (**Figure S3I**), were significantly decreased in the colon tissues of *Cdx2* ^CreERT2^ *Gpx4*^F/F^ mice. The immunoblot analysis of several major antioxidant proteins, including SOD2, NQO1, and HO-1 (Fig. 3C), revealed an increase in these proteins in *Cdx2* ^CreERT2^ *Gpx4*^F/F^ mice, confirming a state of redox imbalance. H&E staining (Fig. 3D) and pathology scoring (Fig. 3E) indicated increased inflammation and tissue damage. While the proliferation marker Ki67 remained unchanged (Fig. 3F and 3G), an increase in the apoptosis marker CC3 was observed in colon tissues of *Cdx2* ^CreERT2^ *Gpx4*^F/F^ mice (Fig. 3H and 3I). This suggests that GPX4 deficiency contributes to enhanced apoptosis during the colitis process. Together, in the absence of vitamin E, mice deficient in GPX4 exhibit heightened susceptibility to DSS-induced colitis.

### GPX4 depletion specifically in colon epithelial cells increases intestinal inflammation in mice treated with a high Mn diet.

**Figure 4:**
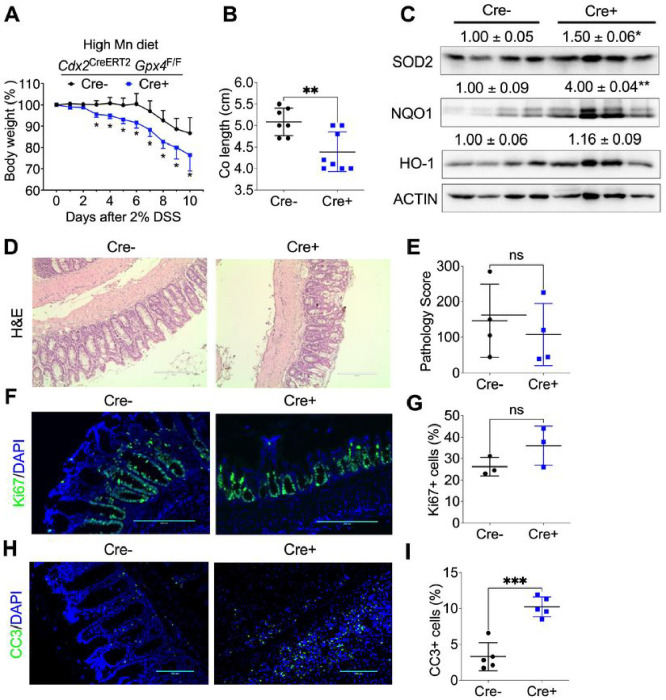
GPX4 depletion specifically in colon epithelial cells increases intestinal inflammation under a high manganese (Mn) diet treatment. **(A)** Body weight loss curve and **(B)** colon length, **(C)** Western blot analysis of antioxidant protein levels of NQO1, HO-1 and SOD2, **(D)** H&E staining and **(E)** pathological score of colon tissues, **(F)** Ki67 staining and **(G)** its quantification, **(H)** CC3 staining and **(I)** its quantification in colon tissues from *Gpx4*^F/F^ (n=7) and *Cdx2* ^CreERT2^ *Gpx4*^F/F^ mice (n=8). *p < 0.05, **p < 0.01, ***p < 0.001, NS not significant. Unpaired Student t test.

While GPX4 is a selenium-dependent enzyme (Weaver et al, 2022), SOD2 is one of the rare mitochondrial enzymes evolved to use Mn as a cofactor over the more abundant element iron (Naranuntarat et al, 2017). Compared with iron, Mn can catalyze the Fenton reaction more effectively to induce higher ROS production (Cheng et al, 2021). We found that when treated with a high Mn diet, *Cdx2* ^CreERT2^ *Gpx4*^F/F^ mice showed more body weight loss (Fig. 4A) and shorter colon lengths (Fig. 4B). Also, there was an increased expression of antioxidant proteins such as SOD2, NQO1, and HO-1 in colon tissues of *Cdx2* ^CreERT2^ *Gpx4*^F/F^ mice compared to *Gpx4*^F/F^ mice (Fig. 4C). However, H&E staining revealed a similar disease score (Fig. 4D and 4E). We found that cell proliferation, as indicated by Ki67 staining, remained unchanged (Fig. 4F and 4G). However, there was an observed increase in apoptosis, demonstrated by CC3 staining in the colons of *Cdx2* ^CreERT2^ *Gpx4*^F/F^ mice (Fig. 4H and 4I). These results indicate that in the presence of a high concentration of Mn, GPX4 deficient mice are more susceptible to DSS-induced colitis.

### GPX4 depletion specifically in colon epithelial cells reduces intestinal inflammation in mice treated with a low Mn diet.

**Figure 5:**
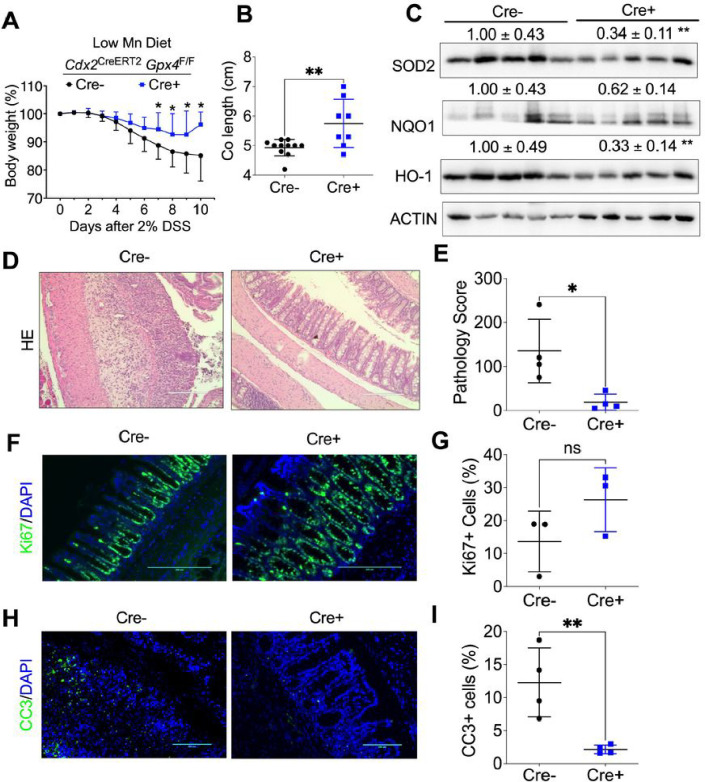
GPX4 depletion specifically in colon epithelial cells reduces intestinal inflammation under a low Mn diet treatment. **(A)** Body weight loss curve, **(B)** colon length, **(C)** Western blot analysis of antioxidant protein levels of NQO1, HO-1 and SOD2, **(D)** H&E staining of colon tissues and **(E)** pathological score, **(F)** Ki67 staining and **(G)** its quantification for colon tissues, **(H)** CC3 staining and **(I)** its quantification in colon tissues from *Gpx4*^F/F^ (n=11) and *Cdx2* ^CreERT2^ *Gpx4*^F/F^ mice (n=8). *p < 0.05, **p < 0.01, NS not significant. Unpaired Student t test.

To test whether Mn is required for enhanced colonic inflammation due to GPX4 deficiency, we treated mice with a low Mn diet. We observed that *Cdx2* ^CreERT2^ *Gpx4*^F/F^ mice experienced less body weight loss (Fig. 5A) and longer colon length (Fig. 5B) than *Gpx4*^F/F^ mice under Mn deficient conditions. Additionally, there was a reduction in the expression of antioxidant proteins such as SOD2, NQO1, and HO-1 in the colon tissue of *Cdx2* ^CreERT2^ *Gpx4*^F/F^ mice (Fig. 5C). H&E staining and pathological scoring revealed reduced inflammation in *Cdx2* ^CreERT2^ *Gpx4*^F/F^ mice when treated with a Mn deficient diet (Fig. 5D and 5E). We also noted Ki67 expression remained unchanged (Fig. 5F and 5G), while reduced apoptosis through CC3 staining in the colons of *Cdx2* ^CreERT2^ *Gpx4*^F/F^ mice (Fig. 5H and 5I), These results indicate that GPX4 depletion reduces intestinal inflammation in mice treated with a low Mn diet.

### Mn deficiency reduces colorectal tumor formation in mice.

**Figure 6:**
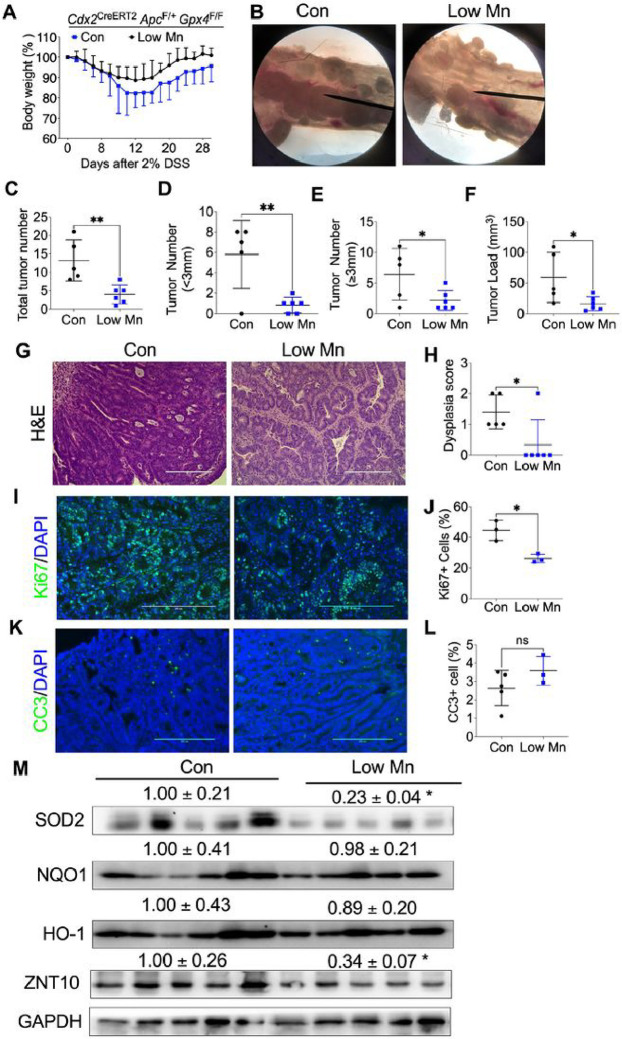
Mn deficient diet reduces colorectal tumor formation. **(A)** Body weight curve, **(B)** macroscopic colorectal tumor images, **(C)** total tumor number, **(D)** tumor number for diameter less than 3mm, **(E)** tumor number for diameter more than 3mm, and **(F)** tumor loading, **(G)** H&E staining and, **(H)** pathological score of colon tumor tissues, **(I)** Ki67 staining in colorectal tumor and **(J)** its quantification, **(K)** CC3 staining in colorectal tumor and **(L)** its quantification. **(M)** Western blot analysis of antioxidant protein levels of NQO1, HO-1 and SOD2 protein expression in colon tissues from *Cdx2* ^CreERT2^ *Apc*^*F/+*^ (n=5) and *Cdx2* ^CreERT2^ *Apc*^*F/+*^ *Gpx4*^F/F^ (n=6) mice. *p < 0.05, **p < 0.01, NS not significant. Unpaired Student t test.

Mn plays a crucial role in intestinal epithelial barrier formation, and a low Mn diet has been linked to an increase in DSS-induced colitis in mice (Choi et al. 2020). However, whether Mn influences the progression of colitis-induced colorectal cancer is not clear. To address this question, we induced colorectal tumor formation under three dietary conditions: a control diet, a Mn-deficient diet, and a high Mn diet. Unfortunately, the high Mn diet was associated with reduced mouse survival during DSS treatment (**Figure S4**). We observed that the Mn-deficient diet did not impact mouse body weight (Fig. 6A) but significantly reduced colorectal tumor formation (Fig. 6B). The Mn-deficient diet group exhibited decreased total tumor numbers (Fig. 6C), smaller tumors (sizes less than 3mm; Fig. 6D), larger tumors (sizes more than 3 mm; Fig. 6E), and overall tumor burden (Fig. 6F). H&E staining revealed lower disease scores in the Mn-deficient diet group (Fig. 6G and 6H). Ki67 staining demonstrated reduced tumor cell proliferation (Fig. 6I and 6J), while CC3 staining showed no change in apoptosis (Fig. 6K and 6L). Western blot analysis indicated reduced levels of the antioxidant protein SOD2 and the Mn efflux protein ZnT10 in the Mn-deficient diet group, while the protein levels of HO-1 and NQO1 remained unchanged (Fig. 6M). Further analysis of metal levels in colon tumors via laser ablation ICP-MS revealed significantly lower Mn levels, but not other metals, in the Mn-deficient diet group compared to the control diet group (**Figure S5A and S5B**). The Mn-deficient diet fed to *Cdx2* ^CreERT2^ *Apc*^*F/+*^ *Gpx4*^F/F^ mice showed a reduction in total tumor numbers (**Figure S6A**) and tumor numbers at size 2–3mm (**Figure S6B**) compared to the control diet. However, it led to no significant differences in smaller tumors (sizes less than 1mm; **Figure S6C**), tumor numbers at size 1–2mm (**Figure S6D**), and larger tumors (sizes more than 3 mm; **Figure S6E**), as well as overall tumor burden (**Figure S6F**). These results suggest that Mn is essential for colorectal tumor formation.

### The SOD mimetic tempol treatment rescues GPX4-inhibited colorectal tumor formation.

**Figure 7:**
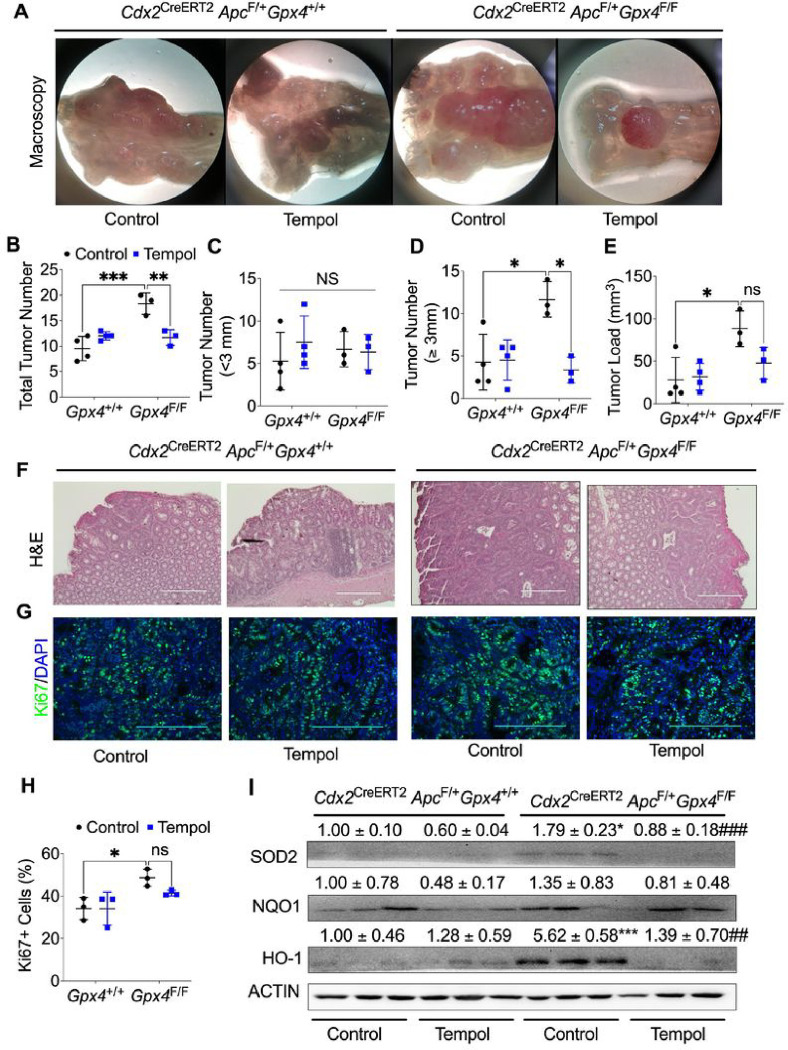
SOD mimetic tempol treatment reduces colon epithelial cells specific GPX4 deficiency enhanced colorectal tumor formation. **(A)** Macroscopic colorectal tumor images, **(B)** total tumor number, **(C)** tumor number for diameter less than 3mm, **(D)** tumor number for diameter more than 3mm, and **(E)** tumor loading, **(F)** H&E staining and **(G)** Ki67 staining and **(H)** its quantification, **(I)** Western blot analysis of antioxidant protein levels of NQO1, HO-1 and SOD2 protein expression of colon tumor tissues from *Cdx2* ^CreERT2^ *Apc*^*F/+*^ (n=7) and *Cdx2* ^CreERT2^ *Apc*^*F/+*^ *Gpx4*^F/F^ (n=7) mice treated with 2% DSS and vehicle control or Tempol. *p < 0.05, **p < 0.01, ***p < 0.001 vs *Cdx2* ^CreERT2^ *Apc*^*F/+*^ mice, ##p < 0.01, ###p < 0.001 vs *Cdx2* ^CreERT2^ *Apc*^*F/+*^ *Gpx4*^F/F^ mice, NS not significant. Two-way ANOVA followed by Tukey’s multiple comparisons test.

To investigate whether GPX4 deficiency-enhanced colorectal cancer depends on oxidative stress, we treated both *Cdx2* ^CreERT2^ *Apc*^F/+^ and *Cdx2* ^CreERT2^ *Apc*^*F/+*^ *Gpx4*^F/F^ mice with the general SOD-mimetic agent tempol. We observed no impact of tempol on mouse body weight (**Figure S7A**); however, under the microscope, *Cdx2* ^CreERT2^ *Apc*^*F/+*^ *Gpx4*^F/F^ mice exhibited increased colorectal tumor formation, a phenomenon that was effectively blocked by tempol treatment (Fig. 7A). Following tempol treatment, *Cdx2* ^CreERT2^ *Apc*^*F/+*^ *Gpx4*^F/F^ mice demonstrated a reduction in total tumor number (Fig. 7B), smaller tumors (sizes less than 3 mm; Fig. 7C), larger tumors (sizes more than 3 mm; Fig. 7D), and overall tumor burden (Fig. 7E). H&E staining further indicated that tempol treatment inhibited the enhanced tumor formation seen in *Cdx2* ^CreERT2^ *Apc*^*F/+*^ *Gpx4*^F/F^ mice (Fig. 7F). Furthermore, tempol treatment effectively inhibited the increased cell proliferation observed in *Cdx2* ^CreERT2^ *Apc*^*F/+*^ *Gpx4*^F/F^ mice (Fig. 7G and 7H) and downregulated the expression of antioxidant proteins, including HO-1 and SOD2 (Fig. 7I). However, CC3 staining revealed no significant change in apoptosis (**Figure S7B and S7C**). In conclusion, our results suggest that GPX4 deficiency may promote colorectal tumor formation by increasing ROS production.

## Discussion

The major findings of our study center around the crucial role of GPX4 in colorectal cancer progression. GPX4, a key player in ferroptosis and cancer, has an unclear role in colorectal cancer. An earlier study reported no significant overall association with colorectal adenoma risk for GPX4 (Peters et al., 2008). However, a recent systematic review showed that carriers of the GPX4 (rs173041) T allele were associated with an increased risk of developing colorectal cancer (Barbosa et al., 2022). Another most recent study reports that high expression of GPX4, which suppresses ferroptosis, was associated with poorer 5-year overall survival only in KRAS mutant tumors from male colorectal cancer patients (Yan et al., 2023). Surprisingly, in our mouse model, GPX4 deletion in colon epithelial cells increased tumor burden. Treatment with tempol suppressed GPX4 deficiency-induced colorectal tumors, highlighting GPX4’s crucial role in inhibiting oxidative stress in colorectal cancer progression.

Oxidative stress, a common feature in various human diseases, including colorectal cancer, is emerging as a significant contributor to colorectal cancer development. In colitis-associated colorectal cancer mice, oxidative stress is heightened in the colon (Lei et al., 2021). GPX4 is the sole enzyme capable of reducing toxic lipid hydroperoxides in biological membranes to the corresponding alcohols using glutathione as the electron donor (Zhang et al., 2021). This aligns with our findings that the deletion of GPX4 resulted in decreased oxidized glutathione levels in colorectal tumors. Consistently, transgenic mice overexpressing GPX4 are protected against oxidative stress-induced apoptosis (Ran et al., 2004). Interestingly, in our study, apoptosis levels were significantly altered in the acute colitis model but not in the colitis-associated colorectal cancer mouse model, suggesting other compensatory apoptotic factors are involved in colorectal tumors.

GPX4 and SOD2 are two of the most important antioxidant defense enzymes that protect cells against oxidative stress (Fan et al., 2011). As a selenoprotein, GPX4 has been reported to play a major role in maintaining the oxidative phosphorylation system and protecting mitochondria from oxidative damage in gut epithelial cells (Cole-Ezea et al., 2012). One study reported significant two-loci interactions between rs4880 (SOD2) and rs713041 (GPX4), reflecting functional interactions between the gene products (Meplan et al., 2010). Deletion of SOD2 in skeletal muscle leads to no major impairment in whole-body metabolism, which is likely partly explained by a compensatory response that may exist from other redox enzymes, including GPX4 (Zhuang et al., 2021). Considering GPX4 deletion didn’t worsen DSS-induced colitis with varied iron diets but showed vulnerability with a vitamin E-deficient diet and increased expression of Mn-dependent SOD2, we propose GPX4 and SOD2 coordinately regulate redox homeostasis.

ROS levels are higher in the ulcerative colitis region, but ROS scavenging enzyme SOD2 is barely detected in resident macrophages, resulting in distinct ROS vulnerability for inflammatory macrophages and resident macrophages (Du et al., 2023). SOD2 upregulation in cancer cells establishes a steady flow of H_2_O_2_ originating from mitochondria that sustains AMP-activated kinase (AMPK) activation and the metabolic shift to glycolysis. Restricting SOD2 expression or inhibiting AMPK suppresses the metabolic switch and dampens the viability of transformed cells, indicating that the SOD2/AMPK axis is critical to support cancer cell bioenergetics (Hart et al., 2015). Also, the absence of SOD2 delays p53-induced tumor formation (Case and Domann 2014). Moreover, overexpression of miR-212 reduces the levels of SOD2 to block the epithelial mesenchymal transition process during colorectal tumor metastasis (Meng et al., 2013). Consistently, we found that antioxidant proteins, especially SOD2, correlated with DSS-induced colitis severity and colorectal tumor numbers in a Mn-dependent manner.

The consumption of antioxidant micronutrients, including Mn, does not modulate the effects of smoking on colorectal cancer risk (Hansen et al., 2013). However, residing in the proximity of industries releasing Mn increases colorectal cancer risk (García-Pérez et al., 2020). In the absence of GPX4, we found a high Mn diet increased susceptibility, while a low Mn diet reduced DSS-induced colitis and significantly decreased colorectal cancer formation. These findings represent conceptual advances in understanding the nuanced impact of GPX4 in colorectal cancer development, shedding light on its role in oxidative stress regulation, particularly in a Mn-dependent manner. The study provides insights into the intricate interplay between GPX4, dietary factors, and their influence on colorectal cancer progression.

In line with our results, hepatocyte restricted GPX4 loss does not suppress hepatocellular tumorigenesis (Conche et al., 2023). In hepatocellular carcinoma, ferroptosis does not provide a cell-autonomous tumor suppressor function but rather triggers an adaptive immune response, placing ferroptosis upstream of CD8 + T cells. Ferroptosis is a potent anticancer target for the treatment of hepatocellular carcinoma and colorectal cancer liver metastasis in combination with immune checkpoint and myeloid-derived suppressor cell blockade, while primary colorectal cancer is resistant to this combinatorial treatment. Our study found that the low Mn diet significantly curtailed colorectal cancer in both GPX4-deficient and wildtype mice. This provides an alternative strategy to treat colorectal cancer.

In conclusion, the study not only unravels the specific mechanisms by which GPX4 influences colorectal cancer but also suggests potential therapeutic strategies. The findings may resonate with researchers and clinicians working in the fields of cancer biology, oxidative stress, and precision medicine. Moreover, the study’s consideration of dietary factors adds a practical dimension, addressing potential preventive measures and highlighting the importance of personalized approaches in cancer care.

## Data Availability

All data are available upon request or through the associated datasets in the manuscript.
